# Changes in Pancreatic Senescence Mediate Pancreatic Diseases

**DOI:** 10.3390/ijms24043513

**Published:** 2023-02-09

**Authors:** Kailin Li, Ji Bian, Yao Xiao, Da Wang, Lin Han, Caian He, Lan Gong, Min Wang

**Affiliations:** 1 College of Food Science and Engineering, Northwest A & F University, Yangling, Xianyang 712100, China; 2 Kolling Institute, Sydney Medical School, Royal North Shore Hospital, University of Sydney, St. Leonards, NSW 2065, Australia; 3 Microbiome Research Centre, St George and Sutherland Clinical School, University of New South Wales, Sydney, NSW 2052, Australia

**Keywords:** pancreas, senescence, morphology, p16^INK4a^, ER stress

## Abstract

In recent years, there has been a significant increase in age-related diseases due to the improvement in life expectancy worldwide. The pancreas undergoes various morphological and pathological changes with aging, such as pancreatic atrophy, fatty degeneration, fibrosis, inflammatory cell infiltration, and exocrine pancreatic metaplasia. Meanwhile, these may predispose the individuals to aging-related diseases, such as diabetes, dyspepsia, pancreatic ductal adenocarcinoma, and pancreatitis, as the endocrine and exocrine functions of the pancreas are significantly affected by aging. Pancreatic senescence is associated with various underlying factors including genetic damage, DNA methylation, endoplasmic reticulum (ER) stress, mitochondrial dysfunction, and inflammation. This paper reviews the alternations of morphologies and functions in the aging pancreas, especially β-cells, closely related to insulin secretion. Finally, we summarize the mechanisms of pancreatic senescence to provide potential targets for treating pancreatic aging-related diseases.

## 1. Introduction

The pancreas is the regulating center of energy consumption and metabolism by secreting digestive enzymes and hormones [1]. The vast majority of the pancreas is exocrine pancreas, and about 95% of the exocrine pancreas consists of acinar cells and duct cells [2,3]. The pancreatic acinar cells produce and release many digestive enzymes, including amylases, lipases, and proteinases, while the ductal cells transport these enzymes into the duodenum [4]. Li et al. also found that adult pancreatic acinar cells might be an important source of β-cell neogenesis [5]. To be specific: (1) the acinar cells are highly polarized. At the base of the cell, the bulk of the endoplasmic reticulum (ER) surround the nucleus, while the zymogen granules containing the digestive enzymes are at the apical part of the cell [6]. (2) The main function of the duct cells is to produce a liquid rich in alkaline bicarbonate. It creates an optimal pH for activating the digestive proenzymes secreted by the acinar cells [7]. (3) The stellate cells contain lipid droplets with vitamin A. They play an important role in producing the characteristic fibrotic matrix in chronic pancreatitis and pancreatic cancer [8]. (4) ATP-sensitive cells are found in intact pancreatic lobules. These immune cells are likely macrophages. In a normal pancreas, the density of these cells are very low, but their number increase significantly in the first days of acute pancreatitis (AP) [6]. The main diseases of exocrine pancreas are pancreatitis and pancreatic cancer. Endocrine cells (α-cells, β-cells, δ-cells, ε-cells, and PP-cells) are spherically clustered into the islet of Langerhans to form the endocrine pancreas [9,10]. β-cells account for more than 85% of the endocrine pancreas [11]. These cells interact with each other and collectively regulate glucose homeostasis [12].

Cell senescence is an irreversible state of cell cycle arrest associated with aging. The aging of different cell types in the pancreas can induce numerous pancreatic diseases. For instance, the acinar cell senescence accelerates AP’s progression. Marta et al. indicated that the incidence of severe AP showed a continuous, linear increase between 20 and 70 years of age up to 9.6%. Meanwhile, patients over 70 years had a19-times higher mortality rate than those under 20 years [13]. Moreover, senescence of the duct cells induces the cancerization of pancreatic tumors, especially ductal tumors, such as pancreatic ductal adenocarcinoma (PDAC) [2]. Also, high-grade pancreatic intraepithelial neoplasia (PanIN) may occur in the aging pancreas or in patients with chronic pancreatitis, but rarely in the healthy pancreas. As for the senescence of endocrine cells, it may lead to damaged proliferation and regeneration of islets that result in pancreatitis, diabetes, and pancreatic neuroendocrine tumors eventually [14]. Notablely, the majority of individuals with type 2 diabetes (T2D) are over 65 years of age [15].

However, there are no unique indicators for the identification of pancreatic senescence. The heterogeneous distribution of exocrine and endocrine cells is not well understood, which may generate many controversies about the changes in and mechanisms of pancreatic senescence. Pancreatic senescence involves many underlying risk factors, including genetic damage, DNA methylation, ER stress, mitochondrial dysfunction, weakening of the immune system, and inflammation [16]. Notably, insulin is originally expressed as proinsulin and its native structure is formed in the ER, which is widely distributed throughout the cytoplasm of pancreatic β-cells [17]. As the disulfide bond connecting the two peptide chains of insulin is mainly formed in the ER, maintaining proper ER proteostasis becomes critical for pancreatic senescence. Indeed, among all factors, impaired protein homeostasis (protein folding, processing, and maturation) has been found to significantly induce pancreatic aging and age-related diseases [18,19]. Moreover, ER dysfunction results in ER stress that accompanies other cellular stresses, including oxidative stress, inflammation, and mitochondrial stress, which crucially induce pancreatic senescence and the development of aging-associated illnesses [17].

Therefore, a deeper understanding of the morphological and molecular changes in the senescent pancreas may help preserve or recover its exocrine and endocrine functions, providing new therapeutic strategies to inhibit the development of pancreatitis, pancreatic cancers, and diabetes in the context of senescence.

## 2. Morphological and Pathological Changes Induced by Pancreatic Senescence

It is widely accepted that the morphology of the pancreas determines its function. Hence exploring age-related morphological and pathological changes in the pancreas can help understand the pathogenesis of pancreatic diseases in the elderly while enhancing a healthy lifespan. As shown in Table 1, the pancreas undergoes various morphological and pathological changes with pancreatic aging, including pancreatic atrophy (especially in the pancreatic tail), hardened texture, main pancreatic ductal dilation, pancreatic lobulation, fatty degeneration, fibrosis, inflammatory cell infiltration, and exocrine pancreatic metaplasia.

### 2.1. General Morphological Changes

Early reports describing alterations in the pancreatic volume were related to autopsy studies in the 20th century, which demonstrated that it decreased significantly with age in healthy persons [20]. More recently, Wang et al. used magnetic resonance imaging (MRI) to investigate the relationships between the age and the anteroposterior diameters of the pancreatic head, body, and tail and pancreatic volume in 226 subjects (112 men and 114 women). All four measurements peaked at the age range of 30–39 years and then gradually decreased with increasing age (*p* < 0.001) [21]. Janssen and Papavassiliou evaluated age-related changes in pancreatic stiffness, wherein semiquantitative elastography showed a significantly stiffened pancreas in healthy individuals over 60 years of age (*p* < 0.001) [22]. To evaluate the increased repeatability of pancreatic stiffness, the scans were repeated in the volunteer on the same day and one month apart by magnetic resonance elastography (MRE) with similar results, showing that pancreatic stiffness significantly increased with age (*p* < 0.001) [23]. In addition, endoscopic retrograde cholangiopancreatography (ERCP) revealed an age-related dilatation of the main pancreatic duct (MPD) [24]. However, age-related dilatation of the MPD does not universally occur in all elderly subjects. Although the majority (63.3%) of elderly subjects aged 70 years or older suffer dilatated MPDs, 31.4% have MPDs of diameters within the defined normal limits, according to Patrick et al. [25]. Notably, most dilatations found by endoscopic retrograde pancreatography (ERP) were global and only a few were confined to the head and/or body [25].

### 2.2. Microstructural and Pathological Changes

Adipose accumulation in the pancreas is also associated with advancing age. Autopsy studies of more than 500 people found that the pancreatic fat content increased in childhood and reached a plateau in middle-aged adults, accompanied by an enhancement in the pancreatic parenchymal volume [26]. Once the participants reached 60 years of age, the volume of their pancreatic parenchyma decreased, leading to a proportionally higher fat content in older adults than in younger ones [26]. The degree of fatty replacement varies both within the pancreas and between individuals. However, the islets of Langerhans and the duct system are usually unaffected. Islets persist as singletons or clusters in the expanding adipose tissue [27,28]. Generally, lipid droplets gradually accumulate in the matrix of the duct epithelial cells, which is named duct epithelial cell fatty degeneration and occurs mainly in the root-tip area of large pancreatic duct epithelial cells [29]. In addition, Petrova et al. stained the mast cells with toluidine blue in Wistar male rats aged 3 months and 18–19 months and showed that the density of granular mast cells in the interlobular region of the pancreas decreased in older rats by approximately 1.5 times [30]. Significantly, this decrease was associated with the overall dampened immune status of the body during aging [30]. Beyond that, there was increased pancreatic focal fibrosis in the elderly. In 89 postmortem specimens from persons without any known pancreatic disease, fibrotic changes were significantly more common in persons older than 60 years; they mainly occurred in the peripheral pancreatic lobes, involving acinar tissue, small ducts, and islets, and were frequently surrounded by lymphocytes [31,32]. Studies in Sprague-Dawley rats also showed that the incidence of islet fibrosis increased in 78-week-old animals, and the incidence of atrophy in the fibrotic islet increased in animals aged over 52 weeks [33]. Moreover, anatomical results also indicated that elderly patients often harbor metaplastic changes in the pancreas, such as acinar to ductal metaplasia, squamous metaplasia, goblet metaplasia, and eosinophil metaplasia [27]. Additionally, some atypical changes may occur during pancreatic aging. For example, because of low estradiol levels in the serum, the onset of spontaneous islet hemorrhage was observed predominantly in elderly men [34]. Although the number of islets and β-cells remained constant or decreased in aged ones, their area may still increase [35]. Amyloid deposition in the islet was also associated with advancing age [36].

### 2.3. Ultrastructural Changes

The ultrastructure of the aging pancreas is generally assessed with a transmission electron microscope (TEM). The acinar cells of old rats showed vacuolation, pyknotic nuclei, uneven distribution of the euchromatins, swollen mitochondria, and broken cristae. The rough ER expanded and showed a scattered arrangement, along with an increase in the number of lipid droplets and lysosomes. There was a significant decreasing trend in the number of zymogen granules. The changes in the nucleus, mitochondria, rough ER, lipid droplets, and lysosomes in islet β-cells were similar to those in acinar cells. Meanwhile, the number of secretory granules in the cytoplasm decreased, accompanied with a larger halo within these particles, which may represent decreased insulin secretion [37]. However, there are different descriptions of secretory granules. For instance, Tuduri et al. thought that inspite of the insulin granule diameter being slightly smaller in aged β-cells, the total number of secretory granules and the secretory vesicle density were similar at different ages. Moreover, although the percentage of mature, dense granules of senescent β-cells may display a modest decrease, the proportion of immature gray secretory vesicles was slightly higher [38]. In addition to differences in the experimental procedures or animal strains, these heterogeneous results may be attributed to the failure in considering the potential effect of age-related changes in peripheral insulin sensitivity on the islet function when analyzing aging process.

**Table 1:** Morphological and pathological changes in pancreatic senescence.

Parts	Variations	Detection Methods	Ref.
**General morphological changes**			
Pancreatic volume	Decrease	Autopsy	[20]
Anteroposterior diameters of the pancreatic head, body, and tail	Peak at the age range of 30–39 years and then gradually decrease	MRI	[21]
Pancreatic stiffness	Increase	Semiquantitative elastography	[22]
	Increase	MRE	[23]
Main pancreatic duct	Dilate	ERCP	[24]
	63.3% dilate (most dilatations are global, a few are confined to the head and/or body); 31.4% is normal	ERP	[25]
**Microstructural and pathological changes**			
Pancreatic fat	Accumulation is accompanied by reduced volume of pancreatic parenchyma	Autopsy	[26]
	Duct epithelial cell fatty degeneration	Autopsy	[29]
	Usually do not affect islets of Langerhans and the duct system	Autopsy	[21]
Density of granular mast cells	Decrease in the interlobular region	Animal model	[30]
Pancreatic focal fibrosis	Increased in 78-week-old SD rats	Animal model	[33]
	Mainly occurred in the peripheral pancreatic lobes, involving acinar tissue, small ducts and islets	Autopsy	[24]
	Lymphocytes encircle	Autopsy	[25]
Harbor metaplastic changes	Acinar to ductal metaplasia Squamous metaplasia Goblet metaplasia Eosinophil metaplasia	Autopsy	[27]
Spontaneous islet hemorrhage	More common in males	Animal model	[34]
Amyloid deposition	In islet	Animal model	[36]
**Ultrastructural changes**			
Nucleus	Vacuolate; Pyknotic; Euchromatins unevenly distribute	TEM	[37]
Mitochondria	Swollen; Cristae broke	TEM	[37]
Rough endoplasmic	Reticulum expand and scatter	TEM	[37]
Quantity of zymogen granules	Decrease	TEM	[37]
Secretory granules of islet cells	Accompanied by a larger halo	TEM	[38]

## 3. Mechanisms of Pancreatic Senescence

As shown in Table 2, pancreatic senescence is caused by many underlying factors, including genetic damage, DNA methylation, ER stress, mitochondrial dysfunction, weakening of the immune system, and inflammation [16]. In addition, β-cell senescence is a dynamic process, which can be accelerated by IR and partially reversed by improved glucose tolerance. For example, Aguayo-Mazzucato et al. showed that removal of aged cells reduced the level of senile markers and senescence-associated secretory phenotype (SASP), while facilitating glucose metabolism and β-cell function [39]. However, it is unclear which steps of β-cell senescence are reversible or irreversible in the context of metabolic stress. Therefore, the details of β-cell senescence’s mechanisms need to be further studied.

### 3.1. Endoplasmic Reticulum Stress

ER stress is the pathological state in which misfolded or unfolded proteins accumulate in the ER due to certain stimuli. Unfolded protein response (UPR) is a specific response to ER stress, which restores protein folding capacity by increasing the synthesis of chaperones (such as 78-kDa glucose-regulated protein, GRP78/Bip). UPR is mainly activated by transmembrane ER stress sensors, including protein kinase RNA (PKR)-like ER kinase (PERK), activating transcription factor 6 (ATF6), and inositol-requiring enzyme 1 (IRE1), which are usually associated with Bip [40]. Of note, UPR also enhances the degradation of unfolded proteins and decreases global protein biosynthesis to maintain intracellular homeostasis [41]. Non-human primates such as cynomolgus monkeys are suitable study models of pancreatic aging because they are similar to humans in terms of pancreatic structure and diabetes susceptibility. Li et al. compared the transcriptomes of islet cells obtained from young and old non-diabetic cynomolgus monkeys using single-cell RNA sequencing [9]. They showed that cell senescence (specifically in β-cells) mainly affected the ER stress and UPR, although gender dimorphism and transcriptional noise existed. The genes involved in age-upregulated transcriptional noise in β-cells were mainly involved in protein folding pathways (such as HSP90B1), protein processing, and maturation. Parallelly, the transcriptomic dysregulation of UPR components was associated with ATF6 and IRE1 signal pathways, however, the PERK branch was not mobilized. The data emphasized that adaptive UPR was activated instead of pro-apoptotic UPR signaling. Notably, in a late aging state, the increased demands for insulin secretion may exceed the secretory capacity of β-cells, leading to a high level of ER stress. Under the circumstances, PERK and pro-apoptotic UPR signaling are activated to deal with the irresolvable ER stress [9]. Therefore, the proliferation and regeneration of senescent β-cells are limited by ER stress, which ultimately reduces insulin secretion, upsets glucose metabolism, and even leads to T2D [42]. These findings suggest the need to find ways to relieve protein homeostasis loss and ER stress in senescent β-cells, which can restore the function of the pancreas.

### 3.2. Mitochondrial Dysfunction

Mitochondrial dysfunction, such as mitochondrial DNA (mt DNA) integrity changes, mitochondrial dynamic (including fusion and fission) abnormalities, and alteration of mitochondrial morphology, as well as the accumulation of damaged mitochondria, are thought to contribute to pancreatic aging [43,44]. In exocrine pancreas, cytosolic Ca^2+^ overloading in AP causes mitochondrial Ca^2+^ overload, which in turn depolarizes the inner mitochondrial membrane, inhibiting the mitochondrial ATP production [45]. However, as the main site of ATP synthesis, mitochondria can regulate both K_ATP_ channels and cytoplasmic Ca^2+^ signals to control insulin secretion in β-cells. The β-cells also respond to intermediate mitochondrial metabolites, such as amino acids, to further enhance insulin release [46]. However, Wortham et al. reported that the mitochondrial number and mt DNA content of the β-cells did not change significantly with age and that no difference in mitochondrial respiration was detected during glucose stimulation or ATP synthase inhibition [47]. Interestingly, the mitochondrial enzymes in β-cells were more abundant with age, promoting the accumulation of TCA metabolites and suggesting that the expression of glucose-stimulated insulin secretion (GSIS)-related metabolic coupling factors, especially malate and NADPH, increased with age [48]. Besides, mitochondria are the major energy provider of eukaryotic cells and the primary source of reactive oxygen species (ROS) at the level of the electron transport chain and targets of protein oxidation [49]. As we know, the generation of free radicals leading to oxidative stress are closely related to elevated ROS levels. The accumulation of damaged macromolecules caused by oxidative stress and protein oxidation is a symbol of cellular senility, which is thought to be involved in a recession of cellular function associated with age [16]. Low testosterone levels together with increased mitochondrial ROS levels were observed in male T2D patients with senescent islet cells, resulting in mitochondrial dysfunction and altered oxidative stress [50]. As a result, age-related mitochondrial damage can lead to pancreatic aging accompanying other complications.

### 3.3. DNA Methylation

Genome-wide studies showed that DNA methylation is significantly altered in the islets of T2D patients [51]. DNA methylation affects the intracellular Ca^2+^ concentration and reduces the expression of the genes controlling β-cell function, which suggests a potential link between pancreatic senescence and T2D [52]. Li et al. showed that age-related DNA methylation inversely correlated with an altered expression of the genes controlling pancreatic function, such as Ras-related protein Rab-3B (*Rab3b*), voltage-dependent L-type calcium channel subunit (*3Cacnb3*), sarcoplasmic/endoplasmic reticulum calcium ATPase 3 (*Atp2a3*), insulin 2 (*Ins2*), among others [51]. *Rab3b* can quickly replenish insulin particles. *3Cacnb3* and *Atp2a3* play an important role in controlling the influx of Ca^2+^ and regulating the membrane Ca^2+^ potential. More importantly, this study reported that the pancreatic exocrine system was more likely to obtain DNA methylation during aging than the endocrine system (*p* < 0.0001). Gene Ontology (GO) analysis showed that the differences in DNA methylation in the pancreatic exocrine system and endocrine system mainly focused on the biological processes such as protein binding, cytoskeleton organization, and amino acid metabolism [53].

### 3.4. Inflammation

The majority of the pancreatic tissue develops a low-level chronic inflammatory state with age. The inflammatory microenvironment of the aging pancreas may be the major cause of the age-dependent deterioration in endocrine function [54]. Kehm et al. reported that advanced glycation end products (AGEs) are formed during normal aging [35]. This causes a proinflammatory response mediated by nuclear factor kappa B (NF-κB) [55]. Proinflammatory reactions accelerate the formation of peroxynitrite, which leads to protein nitrification [56]. Meanwhile, the expression of inflammatory factors in the islets obtained from old and young zebrafish were analyzed. The results showed that the heterogeneous NF-κB signaling becomes preferentially active with age in β-cells [57]. This heterogeneous NF-κB signaling is linked to the differences in the proliferation, regeneration, and function of β-cells, and the high expression of NF-κB signaling in β-cells may reduce the proliferative and regenerative features, causing premature upregulation of *socs2* (an age-related gene that restrains proliferation) [57]. Macrophages also accumulate in the islets during the aging process of zebrafish, leading to the increased expression of TNF-α, which is sufficient to trigger the activation of the NF-κB signaling pathway. An increasing body of evidence suggests that chronic inflammation is a feature of human aging and is related to the dysfunction of β-cells in T2D patients [58].

Several studies have demonstrated the importance of circulating growth factors, such as growth hormone (GH), prolactin (PRL), platelet-derived growth factor (PDGF), and insulin-like growth factor-1 (IGF-1), in age-related proliferation, regeneration, and function of β-cells [59]. Activities of many antioxidant enzymes are reduced in the aged pancreas, which contributes to increased apoptosis [60]. Besides, it was reported that the serine/threonine kinase Akt activation and downstream mammalian target of rapamycin (mTOR) signaling drive pancreatic senescence and are involved in age-related diseases [61]. In addition, changes in the endogenous peptides and overnutrition also accelerate pancreatic aging [52,62]. According to a recent study, the microsomal prostaglandin E synthase-2 (mPGES-2) blockade antagonizes β-cell senescence by acting on NR4A1 [63]. mPGES-2 is one type of prostaglandin E2 (PGE2) synthetase. While not affecting PGE2 production in the liver, kidney, or brown fat, its deficiency reduces the amount of PGE2 in the islets. This finding has great implications for specific recovery of islet senescence. In general, clinicians can prevent premature aging or delay the aging process by understanding the mechanisms of pancreatic aging. Subsequently, a decline in the incidence of diseases associated with pancreatic senescence may follow.

**Table 2:** Potential mechanisms of pancreatic senescence.

Mechanism	Variations	Ref.
ER stress	The genes involved in aging β-cells were mainly concentrated in pathways of protein folding, protein processing, and maturation. In aging β-cells, the transcriptomic dysregulation of UPR components was associated with ATF6 and IRE1 signal pathways, but the PERK branch was not mobilized.	[9]
Mitochondrial dysfunction	The mitochondrial number and mt DNA content of β-cells did not change significantly with age.	[47]
	The mitochondrial enzymes in β-cells were more abundant with age, which promoted the accumulation of TCA metabolites.	[48]
	Increased mitochondrial ROS levels were observed in male T2D patients with senescent islet cells, resulting in mitochondrial dysfunction and altered oxidative stress.	[50]
DNA methylation	The age-related DNA methylation was inversely correlated with altered expression of the genes (such as Rab3b, 3Cacnb3, Atp2a3, and Ins2) controlling pancreatic function.	[51]
	The pancreatic exocrine system was more likely to obtain methylation during aging than the endocrine system.	[53]
Inflammation	The heterogeneous NF-κB signaling becomes preferentially active with age in β-cells of zebrafish, which causes premature up-regulation of socs2 and affects the proliferation, regeneration, and function of β-cells. Macrophages accumulate in the islets during aging, leading to the increased expression of TNF-α.	[57]
Others	Activities of many antioxidant enzymes were reduced in the older pancreas.	[60]
	The serine/threonine kinase Akt activation and its downstream mTOR signaling drove pancreatic senescence and were involved in age-related diseases.	[61]
	The changes in the endogenous peptides and overnutrition accelerated pancreatic aging.	[52,62]

## 4. The Role of Senescence in Types of Pancreatic Diseases

### 4.1. Pancreatitis

Senescence increases the risk of pancreatitis and alters its clinical course, leading to higher mortality rates and therapy costs [64]. Therefore, the course, prevention, treatment, and prognosis of pancreatitis in the elderly have been the focus of many clinical studies.

AP is caused by pancreatic enzyme activation due to various etiologies, such as gallstones, hypertriglyceridemia, metabolic abnormalities, obesity, and alcoholic intemperance [65]. The main pathological feature of AP is the destruction of acinar cells. AP is initiated by an excessive Ca^2+^ signal generation in the acinar cells. There are several reasons for the increase in Ca^2+^ signal generation in the acinar cells: (1) the action of the nonoxidative combination of ethanol with long-chain fatty acids, generating fatty acid ethyl esters; (2) high bile acid concentration in the pancreatic duct; (3) asparaginase-induced intracellular Ca^2+^ elevation; (4) excessive physical pressure. The excessive Ca^2+^ signal production in the acinar cells leads to mitochondrial Ca^2+^ overload, which inhibits mitochondrial ATP generation [6]. Excessive Ca^2+^ signals in the duct and stellate cells also cause AP-like pancreatic characteristic changes. High levels of bile acid (especially taurocholate), nonapeptide bradykinin, proteases, and other transmitters cause major Ca^2+^ signals in the stellate cells [66,67]. In duct cells, a high concentration of acetylcholine can evoke Ca^2+^ signals [6]. Furthermore, ATP and ADP leak out of the damaged acinar cells in the initial stages of AP. Thus, purinergic activation of the macrophages invading the exocrine pancreatic tissue cause Ca^2+^ signals in acinar and stellate cells in response to both ADP and ATP, which promote the release of inflammatory agents [68]. Ca^2+^ overload in primary acinar cells can be prevented or stopped by inhibiting Ca^2+^ release-activated Ca^2+^ channels. This also prevents excessive Ca^2+^ signal production in the stellate cells and macrophages and thus may be an effective treatment of AP [6].

Some studies have linked higher morbidity and mortality of AP with comorbidities in elderly people. Marta et al. indicated that the incidence of severe AP in people aged under 30 years was only 1.6%; however, the incidence of severe AP showed a continuous, linear increase between the ages of 20 and 70 years of up to 9.6%. Meanwhile, patients aged over 70 years displayed a 19-times higher mortality rate than those under the age of 20 years [13]. Moreover, Yang et al. studied the characteristics of acute necrotizing pancreatitis (ANP) in different age stages using MRI. The elderly group exhibited the highest prevalence of ANP (*p* < 0.05) and more frequently suffered from extensive extra-pancreatic involvement such as thrombus formation (*p* = 0.004) [69]. Meta-analysis showed that senescence also influences the clinical course and prognosis of AP [64]. For instance, it was demonstrated that intestinal inflammation during AP is exacerbated in older rats compared with that in younger ones, leading to intestinal barrier dysfunction and increased bacterial translocation [70]. It was also shown that the IL-6 serum levels were higher in aged animals with AP, while the IL-10 serum levels were not statistically elevated. Furthermore, mitochondrial dysfunction was elevated in the aged AP group when compared to that in the young AP group, although it did not deteriorate further than that in the aged non-AP group [71]. In summary, since the aging process may increase the incidence of severe AP, geriatric patients usually have a longer hospital stay and higher mortality than young patients. This finding may be related to the higher expression of inflammatory factors, intestinal barrier dysfunction, and increased bacterial translocation present in the elderly. In addition, mitochondrial dysfunction presented in the elderly patients with AP may be implicated in the enhanced levels of oxidative damage to DNA, proteins, and lipid and the decline of mitochondrial oxidative phosphorylation.

With advancing age, there is progressive pancreatic atrophy and fibrosis, that is, pancreatic senescence, resulting in tissue destruction and chronic pancreatitis (CP) [72]. CP is an irreversible disease of chronic pancreatic fibrosis. Cellular senescence is directly related to the severity of the inflammatory process. Senescent cells are present early in the inflammatory process and largely disappear when inflammation subsides. Fitzner et al. reported that the inflammation, pancreatic stellate cell (PSC) activation, and cellular senescence are timely coupled processes and occur in the same microenvironment of the inflamed pancreas by using a rat model of chronic pancreatitis [73]. Besides, an observational study showed that senescence influences the prognosis of CP and that therapeutic ERCP is safe and feasible in geriatric patients with CP. However, the incidence of moderate to severe complications (including bleeding, infection, perforation, and endoscopic basket impaction) following ERCP increases in elderly patients [74].

### 4.2. Pancreatic Cancer

In addition to tobacco use, heavy alcohol consumption, diabetes, obesity, pancreatitis, high serum vitamin D levels, and radiation, senility is a major risk factor for pancreatic cancer. PDAC originates from the exocrine pancreatic cells and accounts for more than 95% of pancreatic cancer. PDAC is one of the most common malignant tumors in the world, with a 5-year survival rate of about 10%. By 2030, PDAC is projected to become the second leading cause of cancer-related deaths in the United States of America [75]. Moreover, PDAC possesses different molecular characteristics, biological behaviors, and therapeutic responses at different ages.

PDAC mainly affects elderly patients and is rare in those aged <40 years. Several evidences suggest that the predisposition of older individuals to PDAC may be due to the combined pathogenetic effects of a high mutation load, epigenetic dysregulation, telomere dysfunction, weakened stromal reaction, and reduced immunity against the invasion of cancer cells [76]. In addition, the conventional wisdom has it that aged patients cannot withstand aggressive treatments, such as radiotherapy (RT) and chemotherapy (CT), due to chemotherapy intolerance and sensitivity to radiation. For example, Arnold et al. observed larger survival improvements for PDAC patients younger than 75 years at diagnosis than for those aged 75 years and older [77]. Nonetheless, the latest data did not support that increasing RT in young patients improved prognosis or had greater survival benefit than in older patients. However, young people can undergo more extensive surgery and chemoradiotherapy [78,79]. Health conditions such as frailty may be more closely associated with radiation toxicity. Frailty is a pathological state characterized by the decline of various physiological systems, which is related to age but not equal to old age. Therefore, new therapies and technologies are urgently needed for PDAC patients of different ages.

As we know, PDAC can arise from different precursor lesions, such as PanIN, intraductal papillary mucinous neoplasms (IPMNs), mucinous cystic neoplasms (MCN), and possibly, atypical flat lesions (AFL) [75]. The most frequent and characteristic precursor lesion among these is PanIN [80]. It is characterized by microscopic papillary or flattened non-invasive epithelial changes in pancreatic ducts. PanIN can be classified into low-grade lesions (PanIN 1,2) and high-grade lesions (PanIN 3, most likely to transform into carcinoma) according to the cytological and structural changes in the epithelial duct cells. With 40 years of age as the dividing line, PanIN lesions occur more commonly in patients aged over 40 years. Notably, PanIN 3 may occasionally occur in the aging pancreas or in patients with chronic pancreatitis but rarely in the healthy pancreas. Secondly, IPMNs represent about 1% of all pancreatic tumors and 25% of cystic neoplasms [22,81]. According to the World Health Organization, IPMNs grow within the pancreatic ducts and produce mucin [82,83]. IPMNs are divided into four groups based on the degree of atypical hyperplasia and components of invasive carcinoma: low-grade dysplasia of IPMNs, intermediate-grade dysplasia of IPMNs, high-grade dysplasia of IPMNs, and invasive carcinoma of IPMNs [84]. These tumors are also more common in patients with an average age of 65 years. Patients with invasive IPMNs are typically 3–5 years older than those without [28].

It was recently reported that senescence can be triggered by oncogenic signals, including *KRAS* oncogene mutation in pre-malignant lesions such as PanINs, thus serving as a natural barrier to PDAC [85]. In the context of tumors, senescence produces a repressive chromatin state to transcriptionally silence many pro-proliferative genes by a retinoblastoma-dependent program. Moreover, senescence induces the expression of SASP, which can influence the tumor microenvironment (TME) by nuclear factor κB (NF-κB) regulated gene activation program [86]. For example, a study showed that the MEK inhibitor and CDK4/6 inhibitor could lead to the durable cell-cycle exit of *KRAS* mutant pancreas cancer cells through retinoblastoma protein-mediated cellular senescence [87]. Miyasaka et al. examined four markers of senescence (senescence-associated β-galactosidase, senescence-associated heterochromatin foci, p16^INK4a^, and p15^INK4b^), and the results showed that all of them gradually decreased with the progression of IPMN [84]. Therefore, people can clear damaged and pre-malignant tumor cells through cellular senescence-induced proliferative arrest and immune-mediated mechanisms.

As for therapeutic targets of pancreatic cancer, many drugs that target immune and metabolic targets can be considered. In terms of mechanisms, different metabolic regulators, such as lactic acid, phosphoinositide 3-kinase (PI3K), mechanistic target of rapamycin (mTOR), AMP-activated protein kinase (AMPK), *c-Myc*, hypoxia-inducible factor-1α (HIF-1α), p53, and peroxisome proliferator activated-receptors (PPARs), can prevent pancreatic cancer or improve its survival by regulating the metabolism of immune cells and pancreatic cancer cells [88]. Recently, accumulating evidences have found that the microbiome and its products can regulate the pancreatic tumor microenvironment, the biological behavior of pancreatic cancer cells, and the function of the immune system [89]. In addition, type I interferon α (IFNα) suppressed the viability and migration of human pancreatic cancer cell lines while affecting the toll-like receptor (TLR) signaling pathways in human pancreatic cancer cells. So, the IFN and TLR signaling pathways may be therapeutic targets for PDAC [90]. Androgen receptor (AR), expressed in human normal pancreatic tissue and human pancreatic adenocarcinoma tissue, is another candidate for a therapeutic target for pancreatic cancer. Researchers have confirmed that interleukin-6 (IL-6) increased the activation of AR in pancreatic cancer cells by upregulating the phosphorylation of signal transducer and activator of transcription 3 (STAT3) and mitogen-activated protein kinase (MAPK). IL-6 also promoted the migration of pancreatic cancer cells in the presence of AR. AR might also influence the progression of pancreatic cancer by affecting the circadian rhythm [91]. Furthermore, PDAC is closely related to ER stress. Jiang et al. suggested that both GRP78 and poly (ADP-ribose) polymerase (PARP) may have key roles in the chemoresistance of pancreatic cancer. GRP78 might affect many different cellular processes and pancreatic cancer survival and is regarded as one of the valid targets against chemoresistance [40].

### 4.3. Type 2 Diabetes

As an age-related disease, the incidence of T2D in the elderly is much higher than in the adult population (20.2% vs. 10.9%) [92]. The age-dependent increase in T2D prevalence is mediated by the complex interaction of various factors, including reduced physical activity, rising obesity rates, excessive use of hormones and drugs, skeletal muscle loss, and decreased β-cell number and function. Furthermore, changes in the hormonal environment occurring with age, such as a gradual decline in testosterone concentrations in men and a substantial decrease in endogenous estrogen production in women, can also perturb glucose metabolism by reducing insulin secretion and insulin resistance (IR) [93]. In addition, patients with T2D are more likely to develop age-related comorbidities, such as frailty, mild cognitive impairment, Alzheimer’s disease (AD), cardiovascular disease, bladder dysfunction, osteoporosis, visual impairment, and renal dysfunction, indicating that T2D itself may also represent a pro-aging state [94].

During aging, β-cells undergo several metabolic changes. The proliferation and regeneration are limited and the secretory capacity is reduced [35]. Therefore, aging-related degeneration of β-cells contribute to impaired glucose homeostasis and T2D [95]. Specifically, the Genome-Wide Association Study (GWAS) attested that single nucleotide polymorphisms (SNPs) adjacent to the *CDKN2a/b* gene were associated with T2D. It meant that β-cell senescence interrelated genetic defects might aggrandize the susceptibility of T2D [96]. In parallel, it is also widely believed that T2D can promote β-cell senescence. Aging β-cells upregulate the expression of genes associated with aging, including *p21^Cis1^* and *Igf1r*. At the same time, the expression of genes critical to their function and properties (such as *Ins*, *Mafa*, *Pdx1*, and *NeuroD1*) decreased [39]. In conclusion, understanding the genetic changes of β-cells associated with IR in the state of aging may reveal new targets for reversing β-cell senescence, leading to potential T2D treatment by targeting aging β-cells. Yet, the precise mechanisms of T2D associated with the mature or healthy β-cell aging process and premature β-cell senescence are not fully understood. To understand the interaction between β-cell senescence and T2D, both aging characteristics and stress factors / pathways ought to be taken into consideration.

#### 4.3.1. Proliferation and Regeneration of Senescent β-Cells

The proliferation of β-cells is by self-replication, whereas the regenerative capacity of β-cells is by the differentiation of progenitor cells or trans-differentiation of pancreatic non-β cells to β-cells. The proliferative potential of most organs and tissues, including β-cells, declines with age. In parallel, the ratio of β-cells to islet region also decreases [16]. For example, a study on β-cell proliferation markers Ki67 and proliferating cell nuclear antigen (PCNA) in Wistar rats aged 4 months, 14 months, and 24 months showed a significant reduction in β-cell proliferation and an increase in β-cell apoptosis with age [60]. An age-related decline in proliferative potential was also thought to contribute to the increased prevalence of T2D in older adults [97].

**Figure 1:**
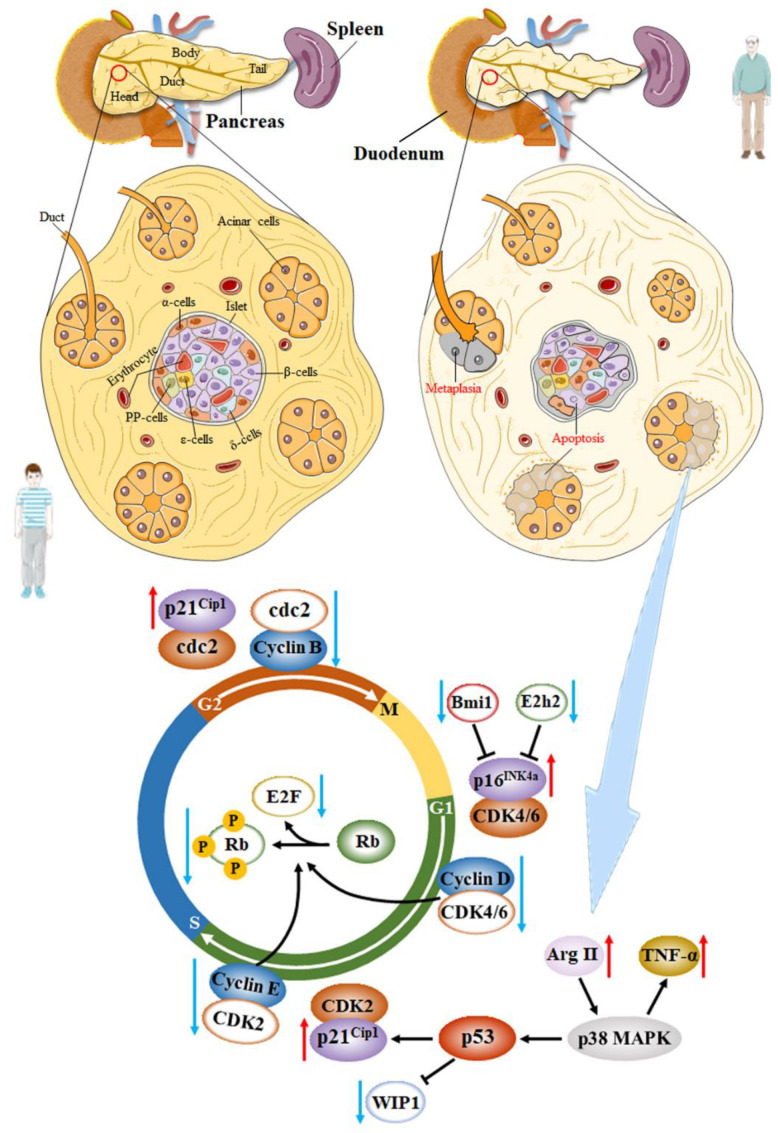
There was a significant reduction in β-cell proliferation and an increase in β-cell apoptosis with age. The p16^Ink4a^ increases with age in islets, inhibiting the phosphorylation of Rb protein by preventing Cdk4/Cdk6 binding to Cyclin D in the early G1 phase. Then the release of E2F transcription factors is blocked and contributes to cell cycle arrest. The increased expression of p16^INK4a^ is associated with the downregulation of Ezh2 and Bmi1. Moreover, the Arginase-II is upregulated in senescent acinar cells and activates the p38 MAPK, leading to the paracrine release of TNF-α and β-cell apoptosis next. The p38 MAPK pathway also alters the levels of WIP1 and p21^Cip1^ induced by p53. The high expression of p21^Cip1^ inhibits the phosphorylation of Rb protein by preventing Cdk2 binding to Cyclin E in the late G1 phase. They may also regulate the cell cycle transition from G2 to the M phase by controlling the combination of cdc2 and Cyclin B.

Multiple studies have demonstrated that the decreased proliferative potential of aging β-cells was associated with decreased cell cycle activators and increased cell cycle inhibitors [98]. It is well known that β-cells express most cell cycle inhibitors, such as p16^INK4a^, p18^INK4c^, p19^Arf^, p21^Cip1^, p27^Kip1^, p53, among others. The role of p16^INK4a^ (encoded by *Cdkn2a*), a tumor suppressor protein and a cyclin kinase inhibitor, in mouse and human β-cell senescence has been widely investigated. p16^Ink4a^ increases with age in both rodent and human islets, inhibiting the phosphorylation of retinoblastoma (Rb) protein by preventing cyclin-dependent kinase 4/6 (Cdk4/Cdk6) binding to Cyclin D in the early G1 phase [47]. Then, the release of E2F transcription factors, controlling the cell cycle transition from the G to the S phase, is blocked and contributes to cell cycle arrest [97,98]. Simultaneously, p16^INK4a^ also participates in β-cell regeneration. Krishnamurthy et al. reported that the p16^INK4a-/-^ mice display much better regenerative capacity following injury of streptozotocin (STZ) compared with those in p16^INK4a+/+^ and p16^INK4a+/−^ mice [99]. Another study showed that the increased expression of p16^INK4a^ in aging β-cells is associated with the downregulation of polycomb proteins Ezh2 and Bmi1 due to the decreased signaling pathway through the PDGF receptor in both mice and humans [100]. However, In *INK4a* deficient mice, β-cell proliferation still declines with age. Some scholars believed that this was related to the expression of p19^Arf^, which was another product of the *INK4a/Arf* locus and was involved in the cell cycle progression by different mechanisms. The function of this protein in β-cells has been less studied. Interestingly, p19^Arf^ is thought to be a direct inhibitor of forkhead Box M1 (FoxM1) in other cell types [99]. Another signal channel associated with proliferation and regeneration decline in aging β-cells is the p38 MAPK pathway, which can alter the levels of phosphatase WIP1 induced by p53 [16]. Xiong and his colleague reported that Arginase-II was upregulated in senescent pancreatic acinar cells and activated p38 MAPK, leading to the paracrine release of Tumor Necrosis Factor (TNF)-α, which caused β-cell apoptosis next [101]. Additionally, the p38 MAPK pathway affects the expression of p53 transcription factors. p21^Cip1^ is the main target of p53. The high expression of p21^Cip1^ can also inhibit the phosphorylation of Rb protein by preventing proliferative kinase Cdk2 binding to Cyclin E in the late G1 phase. However, at the protein level, Hinault et al. did not find differences in p53 expression between the islets of mice aged 3 and 11 months [102]. According to Hinault et al., there was also no significant difference in the expression of p21^Cip1^ in the islets between the 3-month and 11-month mice [102]. Therefore, the roles of p53 and p21^Cip1^ in β-cell senescence need further investigation, as they may also regulate the cell cycle transition from G2 phase to M phase by controlling the combination of cdc2 and Cyclin B (Figure 1).

There are a limited variety of cell cycle activators expressed in β-cells. For example, rodent β-cells only express Cdk4 but not Cdk6. Cyclin D3 is almost undetectable in mouse islets but highly expressed in the human β-cells. However, human β-cells express only a small amount of cyclin D1 and D2 [98]. Moreover, FoxM1 transcription factors regulate the genes involved in cell cycle regulation and cell division. The expression of FoxM1 declines with age in most cell types, including pancreatic islets, which results in reduced β-cell proliferation [103]. Besides, the proliferation and regeneration decline in senescent β-cells of rats and human are also directly associated with a decreased expression of the pancreatic duodenal homeobox 1 (Pdx1) and Ki67 [35]. Nevertheless, it is still possible for β-cells in aged mice to proliferate strongly when exposed to proliferative stimuli such as glucokinase activators, although there is a long quiescent period before they re-access the mitotic cell cycle [59]. This process has been considered to be an adaptive compensation of aging β-cells. However, the studies on proliferation and regeneration during β-cell senescence are still limited and controversial.

#### 4.3.2. Insulin Secretion of Senescent β-Cells

Secreting insulin to maintain glucose homeostasis is a major indicator of β-cell function. The effects of senility on both basal insulin secretion (fasting insulin levels) and glucose-stimulated insulin secretion (GSIS) have been controversial. There are three main theories about the insulin-secreting ability of senescent β-cells: (1) unlike young β-cells, senescent β-cells show an age-dependent decline in GSIS and basal insulin secretion. This theory was thought to be related to the reduction of islet proliferation and regeneration during senescence [104]. (2) Basal insulin secretion does not change significantly or decrease but GSIS increases in aging β-cells, suggesting that insulin secretion in hypoglycemia and hyperglycemia is controlled by distinct mechanisms and can be uncoupled. For example, some studies reported that during normal aging, p16^INK4a^-expressing islets showed improved GSIS, wherein insulin was secreted approximately 2.5-times more under high glucose conditions. However, they did not show enhanced basal insulin secretion [105]. These studies also suggested that both increase and decrease of p16^Ink4a+^ aging β-cells may benefit the pancreatic function [59]. (3) The circulating insulin secretion, both in the fasting state and in response to glucose stress, is higher in older animals, which has been widely reported recently. This viewpoint may also imply that the compensatory metabolic features of the β-cells in response to age-dependent development are impaired glucose tolerance, decreased insulin sensitivity, and elevated IR [35,106,107]. For instance, Helman and his colleagues found that when islets were incubated in high glucose, the β-cells from 6- to 27-months ICR mice secreted more insulin than they did from 1-month mice. In parallel, this situation also happened at low glucose levels [97]. So far, the feature of β-cell adaptation or alteration with age is still a controversial problem. This is partly due to the heterogeneity of endocrine cells, which means that insulin secretion of β-cells from different species or different genders are involved in different mechanisms. Due to the inconsistent expression of senescent cellular markers in different β-cells, some assays may only simulate one aspect of cellular senescence while not fully reflecting the phenotype of β-cell senescence. Besides, individual aging does not necessarily represent β-cell aging. Many studies did not assess the extent of β-cell senescence. A study showed the first stage of insulin secretion was similar between the elderly and the young, but the elderly exhibited a decreased insulin secretion in the second phase [108]. Particularly, a limitation in most studies is that senescent β-cells have been analyzed without considering the potential impact of age-related changes in peripheral insulin sensitivity on their function [38]. These researches suggest that while cellular dysfunction is inevitable during aging, the age-related decline in β-cell function is more likely to be caused by external factors rather than self-directed failures [59]. Therefore, many problems still need to be solved involving how β-cell function alters with age.

**Figure 2:**
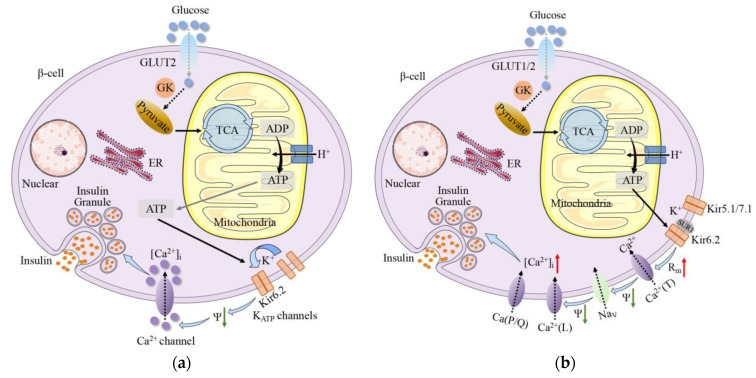
(**a**) The consensus model of GSIS. Glucose is transported into the β-cells via GLUTs. After that, glucose oxidizes and generates ATP, mainly in the mitochondria, through the TCA cycle or the Krebs cycle. Increased ATP/ADP ratio reduces β-cell K_ATP_ conductance, which leads to the depolarization of the cell membrane and initiation of electrical activity. The importance of β-cell electrical activity is that it increases the intracellular Ca^2+^ concentration, which is required to trigger insulin secretion; (**b**) Stimulus-secretion coupling in human β-cells. Glucose is transported into the β-cells via GLUT1 and GLUT2 and leads to accelerated mitochondrial glucose metabolism, increased ATP production, and K_ATP_ channels closure (consisting of the pore-forming subunit Kir6.2 and the sulfonylurea-binding protein SUR1). Increased membrane resistance (R_m_) due to the K_ATP_ channels closure allows the opening of T-type Ca^2+^ channels and a further membrane depolarization. The depolarization activates Na channels and L-type Ca^2+^ channels. At the peak of the action potential, P/Q-type Ca^2+^ channels open and trigger exocytosis of insulin granules. ER: endoplasmic reticulum; R_m_: membrane resistance; [Ca^2+^]_i_: intracellular Ca^2+^ concentration; Ca^2+^(T): T-type Ca^2+^ channels; Ca^2+^(L): L-type Ca^2+^ channels; Ca^2+^(P/Q): P/Q-type Ca^2+^ channels; Na_v_: Na channels.

It is generally believed that there are diverse metabolic steps to modulate GSIS (Figure 2):

(1) Glucose is transported into β-cells via glucose transporters (GLUTs), especially Glut2 which has a low affinity for glucose and a high transport capacity [109]. The research indicated that during aging, the expression of the solute carrier family 2 member 2 (*Slc2a2*) gene for GLUT2 was down-regulated and affected insulin sensitivity [110]. Glut2-related changes in insulin secretion during aging are caused by several factors, such as the level of Sirt1, β-cell sensitivity to incretins, mitochondrial function, and oxidative stress [16]. Additionally, glucose uptake in mice is mediated by GLUT2, while GLUT1 constitutes the primary GLUT in human β-cells [109]. This may lead to different GSIS in human and rodents [60].
(2) After that, glucose metabolism is caused by phosphorylation induced by glucokinase (GK), which is the rate-limiting step in insulin secretion and the first reaction in glycolysis. Actually, almost all the glucose entering glycolysis go into the Krebs cycle [109]. Gong and Muzumdar reported that GK activity significantly increased with age in healthy rats, leading to an increase in GSIS. This situation suggested that β-cells tried to overcome the age-dependent development of impaired glucose tolerance, decreased insulin sensitivity, and elevated IR [60]. Glucose oxidizes and generates ATP in the cytoplasm, mainly in the mitochondria through the tricarboxylic acid (TCA) cycle and Krebs cycle. There is a tight coupling between glycolysis and mitochondrial oxidation. Glycolysis accelerates under the condition of decreasing ATP [111]. Wortham et al. observed that TCA cycle metabolism enzymes or metabolites were more abundant in older mice, which could contribute to the varying GSIS with age [47].
(3) Mice and human β-cells are hyperpolarized (−80 mV vs −70 mV) and electrically silent at low glucose levels [112]. The ATP-sensitive K^+^ (K_ATP_) channel is the main ion channel open at the resting potential in β-cells of all species. Inwardly rectifying K^+^ channels (Kir5.1 and Kir7.1) are also active at lower glucose concentrations, but their contribution to the resting conductance is small [109]. Increased ATP/ADP ratio reduces β-cell K_ATP_ conductance by closing K_ATP_ channels on the cell surface, leading to depolarization of the cell membrane, initiating electrical activity [109]. Gregg et al. proved that p16^INK4a^ down-regulated E2F transcription factors were required for Kir6.2 promoter by inhibiting the phosphorylation of Rb. Kir6.2 was contained in the pore-forming subunits of K_ATP_. Therefore, the high expression of p16^INK4a^ in senescent β-cells reduced K_ATP_ channel activity [113]. Of note, the β-cell must be equipped with an inward current in the absence of other ion channels [109]. The potential candidates for the background inward current include chloride (Cl^−^) channels, transient receptor potential (TRP) channels, and pumps and transporters [114,115].
(4) The importance of β-cell electrical activity is that it increases the intracellular Ca^2+^ concentration, which is required to trigger exocytosis of insulin-containing secretory granules (triggering pathway) [109]. At least in mouse β-cells, the increase in intracellular Ca^2+^ concentration that leads to GSIS is almost entirely due to the influx of extracellular Ca^2+^ through voltage-gated Ca^2+^ channels, with a marginal contribution from intracellular Ca^2+^ storage [116]. In human islets, the depolarization resulting from the T-type Ca^2+^ channel opening activates Na channels and L-type Ca^2+^ channels. At the peak of the action potential, P/Q-type Ca^2+^ channels open and trigger exocytosis of insulin granules [117]. Additionally, mitochondrial metabolism not only leads to ATP production, it also produces necessary coupling factors that amplify insulin secretion, such as glutamate, ATP, and NADPH [118]. Actually, only a few nutrients (such as glucose and leucine) induce insulin secretion on their own. Many other nutrients require the presence of an initiator to promote insulin release [119]. These include most amino acids, fatty acids, hormones, and neurotransmitters, which are referred to as “amplifiers” of insulin secretion [120]. The triggering pathway is necessary, but without the amplification pathway, which primarily affects the sensitivity of the secretory mechanisms, its role is diminished [121]. At the same time, ryanodine receptor (RyR), inositol triphosphate receptor (IP3R), and sarcoplasmic/endoplasmic reticulum Ca^2+^-ATPase (SERCA) in the ER Ca^2+^ pool are involved in the regulation of GSIS by controlling the balance of Ca^2+^ in the ER and cytoplasm [122]. Furthermore, the decrease in Ca^2+^ concentration within the ER can induce store-operated Ca^2+^ entry (SOCE), which is also involved in GSIS [123]. Studies have shown different manifestations of Ca^2+^ in the senescence of β-cells. Some people have announced that they have observed a decline in coordinated Ca^2+^ within human islets during aging, which decreased the GSIS and disrupted the insulin secretion dynamics [124]. Others have suggested that the production of metabolic coupling factors increase during aging. These metabolites can activate the amplifying pathway of Ca^2+^ to enhance GSIS [47].
(5) The enhancement of cytosolic Ca^2+^ triggers the exocytosis of insulin granules. Insulin is stored in the crystalline form in the secretory vesicles as a Zn_2_-insulin_6_ complex [109]. The exocytosis of insulin granules is a multistage process, including vesicle trafficking, docking, and fusing with the plasma membrane [60]. Pclo is one of the key factors in regulating the exocytosis of insulin. Previous studies reported that in the pancreatic tissue, aging increased *Pclo* mRNA levels (*p* < 0.0001) and then showed high insulin levels [110].

## 5. Conclusions

With the continuous application of MRI, ERCP, and other novel in vivo imaging technologies in the anatomy of the pancreas, more and more detailed pancreatic morphological changes are observed at various stages of aging. These morphologic changes in the pancreas also indicate its functional alterations during aging. The decreased expression of cell cycle activators and the elevated expression of cell cycle inhibitors suppress the proliferative and regenerative ability of the aging pancreatic cells. Single-cell sequencing results suggest that HSP90B1 associated with the UPT pathway could be a biomarker of senile β-cells. The analysis of the regulation of GSIS shows that mitochondria, as the main site of ATP synthesis and secretion, also affects islet cells.

β-cell dysfunction determines the severity of diabetes as functional β-cells are essential for controlling glucose homeostasis. As a result, numerous drugs targeting β-cells have been developed to alleviate hyperglycemia and other complications of diabetes. Nevertheless, rather than attenuating and reversing the β-cell senescence, these β-cell-targeted therapies only enhance the function of the remaining β-cells. Animal models have demonstrated that the removal of p16^INK4a^ -positive senescent β-cells mitigated SASP production and improved β-cell function and glucose homeostasis [39, 99]. It is known that ER stress and UPR are closely related to islet cell senescence, especially β-cell senescence. Therefore, targeting ER stress to alleviate β-cell senescence is a potential therapeutic approach for pancreatic aging-related diseases.

## Data Availability

No new data were created or analyzed in this study. Data sharing is not applicable to this article.
